# Sepsisinduzierte Koagulopathie

**DOI:** 10.1007/s00101-026-01748-1

**Published:** 2026-10-21

**Authors:** Martin Mirus, Paul L. Petrick, Thomas Schmoch, Felix C. F. Schmitt, Mirjam Bachler, Lars Heubner

**Affiliations:** 1 Department of Anesthesiology and Intensive Care Medicine, Faculty of Medicine and University Hospital Carl Gustav Carus, TUD Dresden University of Technology, Fetscherstr. 74, 01307 Dresden, Deutschland; 2 Department of Anesthesiology and Intensive Care Medicine, University Hospital Essen, University Duisburg-Essen, 45141 Essen, Deutschland; 3 Department of Anesthesiology and Intensive Care Medicine, Hôpitaux Robert Schuman-Hôpital Kirchberg, 2540 Luxembourg City, Luxemburg; 4 Department of Anesthesiology and Intensive Care Therapy, Philipps-University of Marburg, University Hospital, Marburg, Deutschland; 5 Division of Emergency Medicine and Intensive Care, Department of Internal Medicine, Medical University Innsbruck, Innsbruck, Österreich

**Keywords:** SIC, Blutung, Thrombose, Organversagen, Gerinnung, SIC, Bleeding, Thrombosis, Organ failure, Coagulation

## Abstract

Die sepsisinduzierte Koagulopathie (SIC) stellt eine häufige und prognostisch hochrelevante Komplikation der Sepsis dar, die durch eine fehlregulierte Interaktion zwischen Inflammation, Endothelaktivierung und Gerinnung charakterisiert ist. Pathophysiologisch führen die überschießende Immunantwort auf „pathogen associated molecular patterns“ (PAMPs) und „damage associated molecular patterns“ (DAMPs), die Aktivierung des Komplementsystems, die Bildung neutrophiler extrazellulärer Fallen (NETs) sowie die Schädigung des endothelialen Glykokalyx zu einer prothrombotischen und antifibrinolytischen Mikroumgebung mit Mikrothrombosen, Organfunktionsstörungen und erhöhter Letalität. Etwa 20–25 % der intensivpflichtigen Sepsispatient*innen erfüllen die Kriterien einer SIC. Diagnostisch stehen derzeit verschiedene Scoresysteme wie der ISTH-SIC-, ISTH-overt-DIC- und JSTH-DIC-Score zur Verfügung, deren Aussagekraft jedoch limitiert ist, da sie überwiegend hämorrhagische Parameter erfassen, obwohl die Sepsis typischerweise durch einen thrombotisch dominierten Phänotyp gekennzeichnet ist. Viskoelastische Point-of-Care-Verfahren ermöglichen eine dynamische und bettseitige Beurteilung der Gerinnung einschließlich der häufig vorliegenden Hypofibrinolyse bzw. des „fibrinolytic shutdown“, der mit ungünstigem Outcome assoziiert ist. Für diese spezifische Fragestellung sind die Verfahren bislang jedoch weder ausreichend validiert noch als diagnostischer Standard etabliert. Daten und Empfehlungen zu spezifischen therapeutischen Optionen sind eingeschränkt. Grundlegend ist eine konsequente Thromboseprophylaxe bei Patienten mit Sepsis, wobei niedermolekulare Heparine gegenüber unfraktioniertem Heparin Vorteile hinsichtlich venöser Thromboembolien zeigen. Gerinnungsmodulierende Therapien wie Antithrombin, rekombinantes Thrombomodulin oder aktiviertes Protein C konnten in großen randomisierten Studien bislang keinen konsistenten Überlebensvorteil nachweisen. Experimentelle Ansätze, die auf NETs, die Komplementaktivierung oder die Modulation der Fibrinolyse abzielen, erscheinen mechanistisch vielversprechend, bedürfen jedoch weiterer klinischer Evaluation. Insgesamt erfordert die SIC eine individualisierte, pathophysiologisch orientierte Diagnostik und Therapie.

## Abstract (en)

Sepsis-induced coagulopathy (SIC) represents a frequent and prognostically highly relevant complication of sepsis, characterized by a dysregulated interaction between inflammation, endothelial activation and coagulation. Pathophysiologically, the excessive host immune response to pathogen-associated molecular patterns (PAMP) and damage-associated molecular patterns (DAMP), activation of the complement cascade, formation of neutrophil extracellular traps (NET) and disruption of the endothelial glycocalyx collectively promote a prothrombotic and antifibrinolytic microenvironment, resulting in microvascular thrombosis, organ dysfunction and increased mortality. Approximately 20–25% of critically ill patients with sepsis fulfil the diagnostic criteria for SIC. Current diagnostic approaches rely on scoring systems such as the International Society on Thrombosis and Hemostasis (ISTH)-SIC, ISTH overt-disseminated intravascular coagulation (DIC) and Japanese Society on Thrombosis and Hemostasis (JSTH)-DIC scores; however, their clinical utility remains limited as they predominantly collate hemorrhagic parameters even though sepsis is typically characterized by a thrombotically dominated phenotype. Viscoelastic point-of-care assays enable dynamic bedside assessment of coagulation and fibrinolysis, including the frequently occurring hypofibrinolysis or fibrinolytic shutdown, which is associated with an unfavorable outcome; however, for this specific purpose, these methods have not yet been sufficiently validated or established as a diagnostic standard. Data and recommendations on specific therapeutic options are limited. A consistent pharmacological thromboprophylaxis remains the cornerstone in patients with sepsis, with low-molecular weight heparins demonstrating advantages over unfractionated heparin regarding venous thromboembolic complications. Coagulation modulating treatment, such as antithrombin, recombinant thrombomodulin or activated protein C, have thus far failed to demonstrate consistent survival benefits in large randomized clinical trials. Experimental approaches targeting NET formation, complement activation or fibrinolytic modulation appear mechanistically promising but require further clinical validation. Overall, SIC necessitates an individualized, pathophysiology-driven diagnostic and therapeutic approach integrating inflammatory, endothelial, thrombotic and fibrinolytic alterations.

Nach einer Gewebeschädigung sind die Begrenzung des Blutverlusts und die Verhinderung des Eindringens von Pathogenen in den Blutkreislauf von Bedeutung. Die gleichzeitige Aktivierung von verzahntem Gerinnungs- und Immunsystem gewährleistet beide Funktionen. Unter physiologischen Bedingungen bleibt die Antwort auf geschädigte Gewebe beschränkt, was die Aufrechterhaltung der vaskulären Integrität sowie eine kontrollierte Immunantwort ermöglicht. Charakteristisch für Patienten mit Sepsis ist die generalisierte Aktivierung des Immunsystems [113]. Die beschriebene Verzahnung führt somit auch zur generalisierten Aktivierung des Gerinnungssystems. Die sepsisinduzierte Koagulopathie (SIC) trägt wesentlich zur Organdysfunktion und Mortalität der Sepsis bei [123]. Pathophysiologisch beruht die SIC auf einer sich verstärkenden Interaktion zwischen Inflammation, Endothelaktivierung und Gerinnung, ausgelöst durch die fehlregulierte Immunantwort.

## Definition und Inzidenz

Im Jahr 2001 veröffentlichte die International Society on Thrombosis and Hemostasis (ISTH) einen Score für die Diagnose einer manifesten DIC, der auch für Sepsispatienten gilt (Tab. 1; [117]). Um zu vermeiden, dass eine durch Sepsis verursachte Gerinnungsstörung erst erkannt wird, wenn sie sich klinisch durch Blutungen oder Thrombosen manifestiert, entwickelte eine Arbeitsgruppe der ISTH den 2017 veröffentlichten Sepsis-Induced Coagulopathy(SIC)-Score als frühzeitiges Screeninginstrument [56]. Lyons et al. entwickelten den Sepsis-assoziierten Gerinnungsstörung(SAC)-Score [83]. Als Screeninginstrument liefert der SIC-Score eine dichotome Antwort, der SAC-Score ermöglicht die Unterscheidung zwischen „leichter“, „mittelschwerer“ und „schwerer“ SAC. Sekundäranalysen von klinischen Studien, kamen auf eine SIC-Prävalenz von etwa 20–25 % unter Intensivpatienten mit Sepsis [107, 108]. Die SIC- und die SAC-Diagnose waren mit signifikant erhöhter Mortalität und Morbidität assoziiert [107, 108]. Weitere Erkenntnisse hierzu werden von der 2025 an 59 Intensivstationen in 34 Studienzentren in Deutschland, Österreich und Luxemburg durchgeführten INSIC(„Incidence of Sepsis Induced Coagulopathy“)-Studie erwartet, welche die Inzidenz und Prävalenz der SIC untersucht [109].

**Tab. 1:** Vergleich der Kriterien für sepsisassoziierten Koagulopathien. (ISTH-Kriterien aktualisiert nach Iba et al. 2025 [60]; SAC-Klassifikation nach Schmoch et al. 2025 [107])

Parameter	Punkte	ISTH overt DIC Update 2025 [60]	ISTH SIC [56, 60]	SAC-Klassifikation [107]
Voraussetzung	–	DIC-assoziierte Grunderkrankung	Sepsis	Sepsis
Thrombozytenzahl (× 10^9^/l)	0	≥ 100	≥ 150	**Mild** 1,2 ≤ INR < 1,4 *und* 100 < TZ ≤ 150 **Moderat***:* 1,4 ≤ INR < 1,6 *oder* 80 ≤ TZ ≤ 100 **Schwer** INR ≥ 1,6 *oder* TZ ≤ 80
	1	50 bis < 100	100 bis < 150	
	2	< 50	< 100	
PT-Verlängerung/INR^a^	0	< 3 s	≤ 1,2	
	1	3 bis < 6 s	> 1,2 bis ≤ 1,4	
	2	≥ 6 s	> 1,4	
Fibrinogen (g/l)	0	≥ 1,0	–	
	1	< 1,0	–	
D‑Dimer^b^	0	≤ 3 × OGN	–	
	2	> 3 bis ≤ 7 × OGN	–	
	3	> 7 × OGN	–	
SOFA-Summe^c^	0	–	0	
	1	–	1	
	2	–	≥ 2	
Bewertung	–	≥ 5 Punkte: vereinbar mit manifester DIC	≥ 4 Punkte *und *Thrombozyten- + INR-Subscore > 2: SIC-Kriterien erfüllt	Keine Punktsumme

ᵃ ISTH overt DIC: Verlängerung der PT gegenüber dem Kontrollwert in Sekunden; SIC: INR.

ᵇ OGN = obere Grenze des assayabhängigen Normbereichs. Es gilt die höchste zutreffende Punktzahl.

ᶜ Summe der SOFA-Subscores für Atmung, Kreislauf, Leber und Niere; maximal 2 Punkte im SIC-Score.

ᵈ SAC ist eine Schweregradeinteilung und wurde durch das ISTH-Update 2025 nicht revidiert.

– nicht Bestandteil des Scores bzw. keine Punktezuweisung. Ein ISTH-overt-DIC-Score < 5 schließt eine frühe DIC nicht aus.

*DIC* disseminierte intravasale Koagulopathie, *INR* International Normalized Ratio, *ISTH* International Society on Thrombosis and Haemostasis, *PT* Prothrombinzeit, *SAC* sepsisassoziierte Koagulopathie, *SIC* sepsisinduzierte Koagulopathie, *SOFA* Sequential Organ Failure Assessment, *TZ *Thrombozytenzahl (× 10^9^/l, entsprechend 1/nl)

## Pathophysiologie der SIC

Die fehlgeleitete Wirtsantwort auf charakteristische molekulare Strukturen von Mikroorganismen („pathogen associated molecular patterns“ [PAMPs]), die vom angeborenen Immunsystem als fremd erkannt werden, oder die Wirtsantwort auf aus körpereigenen, geschädigten oder nekrotischen Zellen freigesetzte Gefahrensignale („damage associated molecular patterns“ [DAMPs]) führen zu einer ausgeprägten Hyperinflammation. Die rasche Freisetzung proinflammatorischer Zytokine und Chemokine („Zytokinsturm“) durch Monozyten, Makrophagen, Neutrophile und dendritische Zellen unterhält sich durch die Mediatorfreisetzung aus rekrutierten Immunzellen selbst [5, 35, 63, 139, 141]. Zytokine wie TNF‑α und IL-1β aktivieren Endothelzellen, erhöhen die Expression von Adhäsionsmolekülen und fördern zusätzlich die Freisetzung proinflammatorischer und prokoagulatorischer Faktoren. Mikrothromben entstehen, die den Entzündungsprozess weiter antreiben [17–19]. Inflammation und Gerinnungsaktivierung verstärken sich somit gegenseitig [77].

Die ausgeprägte Aktivierung des Komplementsystems in der Sepsis führt zur massiven Freisetzung von C3a, C5a und dem terminalen Komplementkomplex (C5b-9) und trägt wesentlich zur Pathophysiologie der Sepsis bei. Insbesondere C5a, ein hochpotenter Anaphylatoxin-Mediator, induziert endotheliale Dysfunktion, erhöhte Gefäßpermeabilität, Neutrophilenrekrutierung sowie eine ausgeprägte proinflammatorische Immunantwort [28, 43, 96]. Anhaltend erhöhte C5a-Spiegel sind bei schwerer Sepsis mit Immunparalyse, bakterieller Clearance-Störung und Multiorganversagen assoziiert und begründen C5a als potenzielles therapeutisches Ziel [33, 40].

Komplementfragmente aktivieren Neutrophile und Monozyten [40]. Im Rahmen der Erregerbekämpfung kommt es zum programmierten Zelltod von neutrophilen Granulozyten, was nachfolgend zu einer Geflechtbildung (NETose) aus DNA, Histonen (basische Proteine, um die sich die DNA windet) und den Enzymen neutrophiler Elastase und Myeloperoxidase führt: „Neutrophil extracellular traps“ (NETs) entstehen. NETs können Pathogene über ihre Netzstruktur binden und eliminieren. Jedoch aktivieren NETs gleichzeitig die Gerinnung über Plättchenaktivierung sowie FXII-Aktivierung durch extrazelluläre DNA und Histone, induzieren TF sowie die Hemmung natürlicher Antikoagulanzien und verändern die Fibrinstruktur mit Hemmung der Fibrinolyse [3, 13, 17, 20, 30, 51, 82, 111, 114, 126, 134].

Die Schädigung des endothelialen Glykokalyx, einer zentralen Barriere für die vaskuläre Homöostase, ist mit Krankheitsschwere, dem Auftreten einer DIC, Organversagen und erhöhter Sterblichkeit verbunden [9, 14, 27, 41, 54, 61, 101, 105]. Glykokalyx-Shedding wird durch inflammatorische Mediatoren, Endotoxine, NETs, oxidativen Stress und möglicherweise durch exzessive Flüssigkeitsgabe verstärkt [52]. Der Verlust des Glykokalyx legt Rezeptoren und Adhäsionsmoleküle frei, fördert u. a. so die Leukozytenadhäsion, führt zu Mikroperfusionsstörungen sowie Endotheldurchlässigkeit und zu Gewebsödemen [7, 21, 115]. Aktivierte Endothelzellen verstärken dies durch die Freisetzung von „tissue factor“, vWF, PAI‑1 und aktiviertem TAFI, was das Gleichgewicht in Richtung Prokoagulation und Antifibrinolyse verschiebt. Auch Thrombozyten und ihre extrazellulären Vesikel, reaktive Sauerstoff- und Stickstoffspezies tragen zur Endothelschädigung bei [23, 64, 65].

Sowohl der Tissue-Factor-Weg (extrinsisch) als auch der Kontaktweg über FXII (intrinsisch) tragen wesentlich zur gegenseitigen Potenzierung von Entzündung und Gerinnung bei [36–39]. Entzündungsmediatoren erhöhen die TF-Expression insbesondere auf Endothelzellen und Monozyten, und in der Sepsis ist TF ein starker Prädiktor für die Kurzzeitletalität [11, 93, 127, 137]. FXII kann durch PAMPs, DAMPs sowie durch künstliche Oberflächen wie Katheter oder extrakorporale Kreisläufe aktiviert werden. Auch NETs tragen durch die FXII-Aktivierung und Tissue-Factor-Freisetzung eine zentrale Rolle [34, 100, 131, 137].

Aktivierung und Verbrauch von Gerinnungsfaktoren und Thrombozyten können zur Koagulopathie mit Blutungen führen [97]. Auch antikoagulatorische Faktoren wie Antithrombin und Protein C werden konsumiert, während Thrombomodulin infolge Endothelschädigung vom Endothel abgespalten und in löslicher Form freigesetzt wird [2, 88, 94, 112]. Dieses Zustand entspricht der DIC und ist mit schlechter Prognose assoziiert [37, 78].

**Abb. 1:**
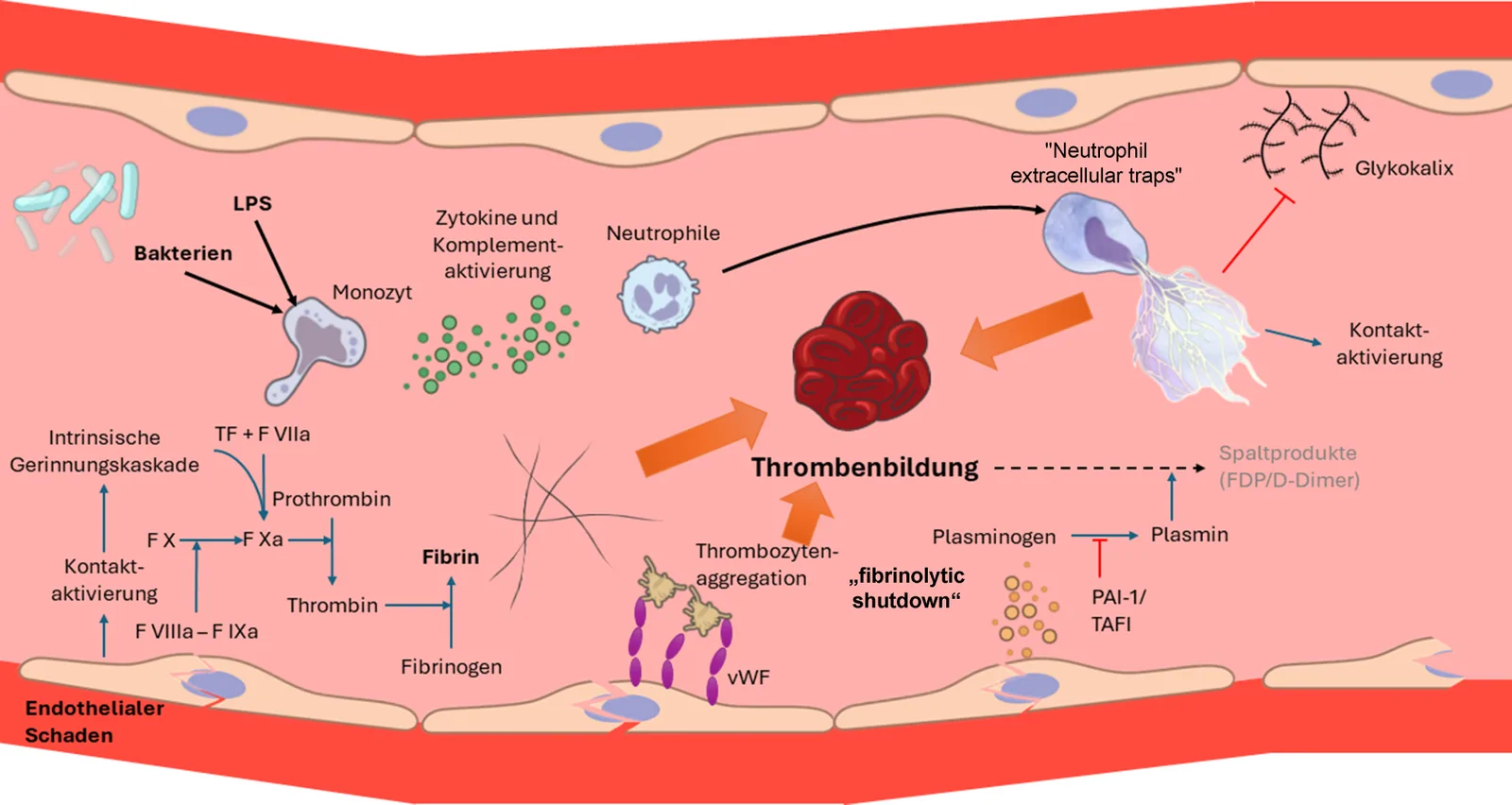
Überblick über die zentralen Mechanismen der Pathogenese der sepsisinduzierten Koagulopathie. *F* Faktor, *LPS* Lipopolysaccharid, *PAI‑1* Plasminogen-Aktivator-Inhibitor 1, *TAFI* Thrombin-aktivierter Fibrinolyseinhibitor, *TF* „tissue factor“, *vWF* von-Willebrand-Faktor. (Die Erlaubnis zur Verwendung der dargestellten Abbildungen erfolgte im Rahmen einer bestehenden Creative Commons 0 1.0 Lizenz [https://creativecommons.org/publicdomain/zero/1.0/] oder einer bestehenden Creative Commons BY 4.0 Lizenz [https://creativecommons.org/licenses/by/4.0/])

Der Überschuss antifibrinolytischer Faktoren wie aktiviertem TAFI (aTAFI) oder PAI‑1 sowie das Auftreten fibrinolyseresistenter Fibrinstrukturen können in der Sepsis zur Hypofibrinolyse bis zum „fibrinolytic shutdown“ führen. Hypofibrinolyse und „fibrinolytic shutdown“ sind mit erhöhter Krankheitsschwere, thromboembolischen Komplikationen, Organfunktionsstörungen und Letalität verbunden [20, 70, 71, 118]. Insbesondere persistierende Mikrothrombosen tragen zur Organdysfunktion und zu schlechterem Outcome bei [118]. Die Abb. 1 gibt eine Übersicht über verschiedene relevante Mechanismen der SIC.

## Diagnostik und Nutzen von POC-Verfahren

Aufgrund der fehlenden einheitlichen Definition der sepsisinduzierten Koagulopathie variieren die diagnostischen Kriterien zwischen den Fachgesellschaften. Dies führt zur unterschiedlichen Gewichtung thrombotischer und fibrinolytischer Parameter (Tab. 2) innerhalb der verschiedenen Bewertungssysteme (Tab. 3; [19]). Angesichts des bei Sepsis häufig dominierenden thrombotischen Phänotyps erscheinen Diagnose-Scores mit Berücksichtigung thrombotischer Parameter zumindest aus theoretischen Erwägungen plausibler [46].

**Tab. 2:** Übersicht relevanter Parameter zur Charakterisierung der sepsisinduzierten Koagulopathie (SIC) nach pathophysiologischen Bereichen. Der Thrombozyten-Pathway umfasst Thrombozytenzahl, ADAMTS13 und vWF. Die Prokoagulationsaktivität wird über TF, F1.2, TAT und Fibrinogen erfasst. Die Fibrinbildung wird anhand löslicher Fibrinmonomere beschrieben. Die natürliche Antikoagulation wird durch APC, APS, TFPI, AT III und TM abgebildet. Die Fibrinolyse umfasst VET, PAP, PAI‑1, TAFI, Plasminogen, FDP/D-Dimer sowie tPA

Pathway	Parameter
Thrombozyten	Thrombozytenzahl; ADAMTS13; vWF
Prokoagulation	TF; Prothrombinfragmente F1.2; TAT; Fibrinogen
Fibrinbildung	Lösliche Fibrinmonomere
Antikoagulation	APC; APS; TFPI; AT III; TM
Fibrinolyse	VET; PAP; PAI‑1; TAFI; Plasminogen; FDP/D-Dimer; tPA

*ADAMTS13* A „disintegrin and metalloprotease with thrombospondin type 1 motif, member 13“, *vWF* von-Willebrand-Faktor, *TF* „tissue factor“, *F1.2* Prothrombinfragment 1.2, *TAT* Thrombin-Antithrombin-Komplexe, *APC* Activated Protein C, *APS* Activated Protein S, *TFPI* „tissue factor pathway inhibitor“, *AT III* Antithrombin III, *TM* Thrombomodulin, *VET* viskoelastische Tests, *PAP* Plasmin-Antiplasmin-Komplexe, *PAI‑1* Plasminogen-Aktivator-Inhibitor 1, *TAFI* Thrombin-aktivierbarer Fibrinolyseinhibitor, *FDP* Fibrin‑/Fibrinogenabbauprodukte, *tPA* „tissue-type plasminogen activator“

**Tab. 3:** Vergleich verschiedener Diagnose-Scores für die DIC, die auch im Rahmen der Sepsis genutzt werden. Gegenübergestellt sind die inkludierten Parameter der JAAM-2-DIC-Kriterien, der ISTH-overt-DIC-Kriterien sowie der JSTH-Kriterien. Aus Gründen der Übersichtlichkeit wurden die einzelnen Grenzwerte innerhalb der Scores nicht dargestellt. Berücksichtigt werden PT-Ratio bzw. PT, Fibrinogen, FDP, TZ sowie – in den JSTH-Kriterien zusätzlich – AT III. Unterschiede bestehen insbesondere hinsichtlich der Einbeziehung der Ätiologie (nur JSTH) und der Gewichtung einzelner Marker

JAAM-2-DIC [129]	ISTH-overt-DIC	JSTH-DIC [57]
–	–	Ätiologie
PT-Ratio	PT	PT
–	Fibrinogen	Fibrinogen
Fibrinabbauprodukte	FDP	FDP
TZ	TZ	TZ
–	–	AT III

*JAAM* Japanese Association for Acute Medicine, *ISTH* International Society on Thrombosis and Haemostasis, *JSTH* Japanese Society on Thrombosis and Hemostasis, *DIC* disseminierte intravasale Koagulopathie, *PT* Prothrombinzeit, *PT-Ratio* Verhältnis der gemessenen PT zur Norm, *FDP* Fibrin‑/Fibrinogenabbauprodukte, *TZ* Thrombozytenzahl, *AT III* Antithrombin III

Der verbreitete ISTH-overt-DIC-Score steigt mit Veränderungen, die ein erhöhtes Blutungsrisiko anzeigen [58]. Der modifizierte JSTH-DIC-Score berücksichtigt teilweise das antikoagulatorische Potenzial des Gerinnungssystems [57]. Die Sterblichkeit nahm linear mit der Höhe des Scores zu und stieg von 23 % (1 bis 3 Punkte) auf bis zu 53 % (7 Punkte) [57].

**Abb. 2:**
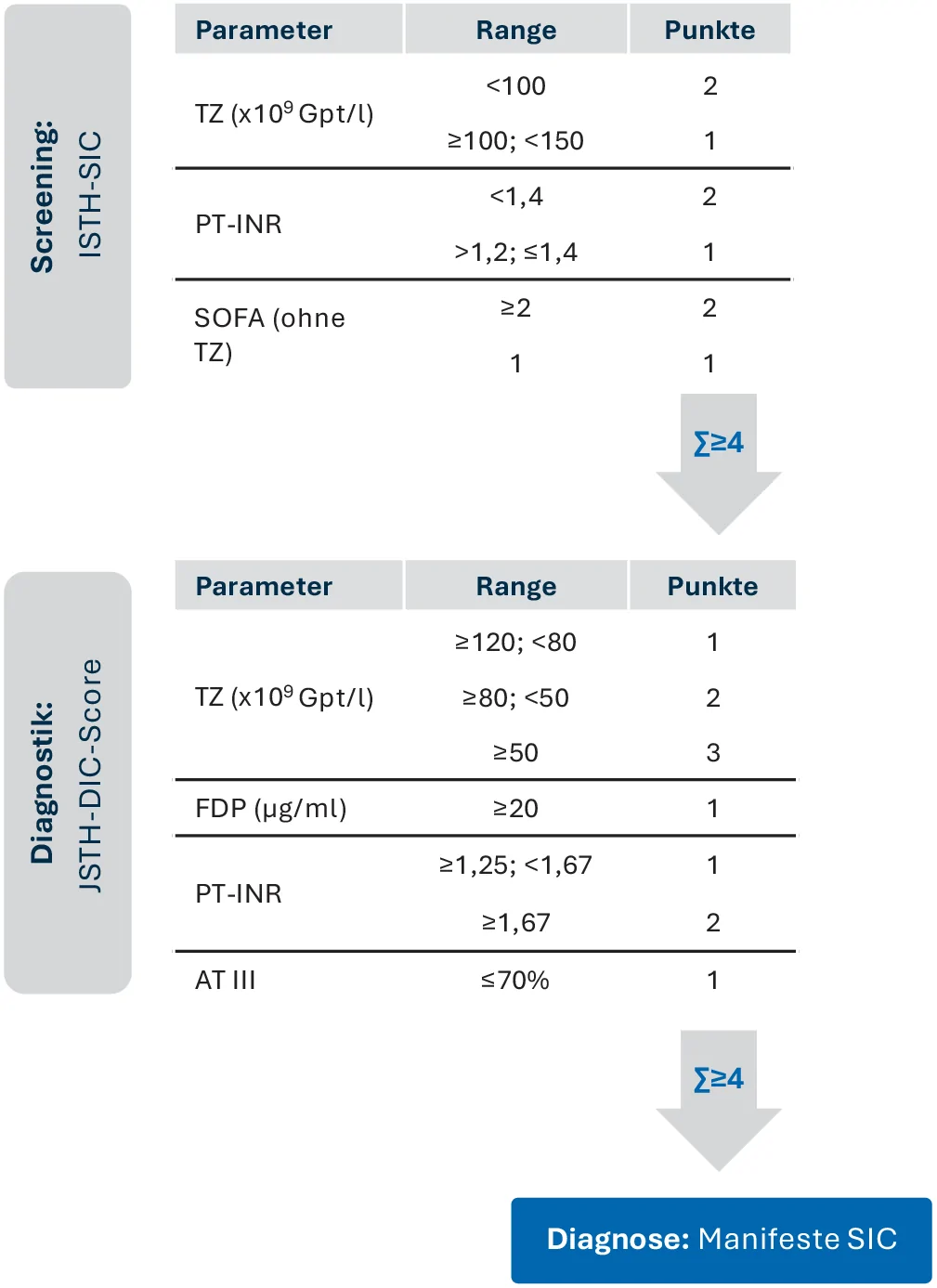
Die Abbildung zeigt ein zweistufiges diagnostisches Vorgehen zur Identifikation einer manifesten sepsisinduzierten Koagulopathie (SIC). Im ersten Schritt erfolgt das Screening anhand des ISTH-SIC-Scores. Ein Score von ≥ 4 Punkten weist auf eine relevante SIC hin und führt zur weiterführenden Diagnostik mittels JSTH-DIC-Scores. Auch hier gilt ein Schwellenwert von ≥ 4 Punkten als diagnostisch wegweisend. Erreichen beide Score-Systeme jeweils den definierten Cut-off, wird die Diagnose einer manifesten SIC gestellt. *TZ* Thrombozytenzahl, *PT-INR* International Normalized Ratio der Prothrombinzeit, *SOFA* Sequential Organ Failure Assessment, *FDP* Fibrin‑/Fibrinogenabbauprodukte, *AT III* Antithrombin III, *ISTH* International Society on Thrombosis and Haemostasis, *SIC* sepsisinduzierte Koagulopathie, *JSTH* Japanese Society on Thrombosis and Hemostasis, *DIC* disseminierte intravasale Koagulopathie

Die vorgelagerte Anwendung von Screeningtools kann die Effizienz diagnostischer Scores steigern. Ein verbreitetes Screeninginstrument ist der ISTH-SIC-Score [56], ein pragmatisches Tool, das in einer retrospektiven Analyse eine Sensitivität von 74,3 % und eine Spezifität von 37,1 % zur Letalitätsvorhersage zeigte [22]. Bei positivem Screening kann der JSTH-DIC-Score angeschlossen werden (Abb. 2). Für die Validität dieser Testkombination liegen jedoch keine Daten vor. Eine prospektive Studie untersuchte die Kombination des ISTH-SIC-Scores als Screening mit anschließenden DIC-Diagnose-Scores (JAAM-DIC und ISTH-overt-DIC) und fand keine Verbesserung der Sensitivität oder Spezifität zur Vorhersage der 7‑Tages-Letalität [47]. Der positive Vorhersagewert (PPV) lag mit und ohne vorgeschaltetes Screening bei 31–38 %. Der SIC-Score allein zeigte in dieser Studie einen PPV von 18 %. Für die Kombination des ISTH-SIC-Screenings mit dem modifizierten JSTH-DIC-Score existieren bislang keine Daten.

Zukünftig sollten neben der Validierung sinnvoller Screening-Diagnose-Score-Kombinationen auch die in den Scores enthaltenen Parameter weiter geprüft werden. Das Gros der Scores fokussiert auf ein hämorrhagisches DIC-Profil, dessen Relevanz in der Sepsis fraglich ist [46]. Neben der stärkeren Einbeziehung antikoagulatorischer Parameter (Tab. 2) könnte insbesondere die Integration viskoelastometrischer Tests (VET) die Diagnostik und Risikostratifizierung bei Sepsis verbessern. VET ermöglichen eine bettseitige Erfassung einer reduzierten oder aufgehobenen Fibrinolyse [50]. Ihre Anwendung zur Beurteilung sepsisassoziierter Fibrinolysestörungen bedarf jedoch noch einer weiteren Standardisierung und klinischer Validierung. Die zugrunde liegende Störung der Fibrinolyse muss in der Sepsis nicht homogen sein; möglicherweise können modifizierte VET-Assays individuell vorherrschende Pathomechanismen identifizieren [18]. Sowohl ein vollständiger Fibrinolyse-Shutdown als auch eine gehemmt verlaufende Fibrinolyse sind mit einer erhöhten Sterblichkeit assoziiert, was eine Berücksichtigung in zukünftigen Scores nahelegt [1, 49]. Eine Metaanalyse zu VET bei Sepsis zeigte, dass ein hypokoagulatorisches Muster (TEG® [Haemonetics, USA]: MA < 51 mm; ROTEM®: MCF-INTEM < 50 mm) sowie eine verringerte maximale Clotfestigkeit im EXTEM mit erhöhter Letalität vergesellschaftet waren [12]. Limitiert wird die Aussagekraft dieser Analyse durch die hohe Heterogenität, die fehlende systematische Erfassung der Lyseparameter sowie die nicht definierte zeitliche Einordnung der VET-Messungen im Sepsisverlauf.

## Thromboseprophylaxe bei Patienten mit Sepsis

Zur pharmakologischen Thromboseprophylaxe werden bei septischen Patienten v. a. niedermolekulare Heparine (NMH) und unfraktioniertes Heparin (UFH) eingesetzt. Eine aktuelle Metaanalyse zeigte eine signifikant geringere VTE-Inzidenz bei intensivpflichtigen Patienten, die NMH im Gegensatz zu UFH erhielten, bei vergleichbarer Rate schwerer Blutungskomplikationen. Dieser Vorteil wird auf die bessere Bioverfügbarkeit und längere Halbwertszeit von NMH zurückgeführt [25, 29, 136]. Bei eingeschränkter Nierenfunktion können NMH akkumulieren. Erfolgt der Einsatz von NMH bei schwerer Niereninsuffizienz sollten ein engmaschiges Monitoring mittels Anti-Xa-Spiegel und entsprechende Dosisanpassungen erfolgen [38].

Neben der medikamentösen Prophylaxe wurde auch die Wirksamkeit mechanischer Maßnahmen, insbesondere der intermittierenden pneumatischen Kompression (IPC), untersucht. Zwei systematische Übersichtsarbeiten zeigten eine signifikante Reduktion von VTE im Vergleich zu keiner Prophylaxe, ohne Einfluss auf die Gesamtletalität [42, 124]. Auch wenn die Datenlage zur mechanischen Thromboseprophylaxe widersprüchlich ist, deutet die aktuelle Evidenz auf einen potenziellen Nutzen von IPC hin, wenn eine pharmakologische Prophylaxe nicht möglich ist.

## Therapeutische Optionen der SIC – Wo stehen wir heute?

### Antithrombin (AT)

Beim Menschen sind niedrige AT-Spiegel während der Sepsis mit höherer Mortalität und ausgeprägterer Organdysfunktion assoziiert, was eine AT-Substitution nahelegt. Einige Studien führten zu Verbesserungen der Hämodynamik, einer Reduktion inflammatorischer Marker sowie einer deutlicheren Erholung der Gerinnungsparameter – ohne relevante Zunahme des Blutungsrisikos [36, 59, 62, 94, 132]. Ältere klinische Studien deuten auf Vorteile bezüglich der Organdysfunktion sowie das Überleben bei Sepsis und DIC durch die Gabe von AT hin [36, 62, 132]. Diese Daten bildeten die Grundlage der KyberSept-Studie, der bis heute wichtigsten Untersuchung zur AT-Therapie in der Sepsis.

In KyberSept erhielten Patient*innen mit schwerer Sepsis entweder hoch dosiertes AT oder Placebo [125]. In der Gesamtkohorte zeigte die Studie keinen signifikanten Überlebensvorteil. Bei gleichzeitiger Heparintherapie war die AT-Gabe mit vermehrten Blutungen verbunden [125]. Patienten *ohne* begleitende Heparinbehandlung wiesen einen signifikanten Überlebensvorteil durch AT auf [69]. Möglicherweise beeinträchtigte die Heparintherapie antiinflammatorische Signalwege und den Schutz des Endothels bzw. positive Glykokalyx-Effekte von AT. Damit erscheint AT bei ausgewählten Patienten, insbesondere ohne parallele Heparintherapie, potenziell wirksam.

Kleinere Studien unterstützten die Theorie, dass AT antiinflammatorisch wirkt und die Organfunktion und Mikrozirkulation in bestimmten Patientengruppen verbessert. Bei ECMO-Patienten war die AT-Supplementierung mit einem schnelleren Abfall von IL‑6, IL-1β und TNF‑α verbunden und damit konsistent mit den antiinflammatorischen Eigenschaften von AT [98]. Diese widersprüchlichen Ergebnisse zeigen: Für gerinnungsmodulierende, antiinflammatorische SIC/DIC-Therapien mit potenziellem Blutungsrisiko sind sorgfältige Patientenselektion und individualisierte, zielgerichtete Anwendung (Zielspiegel vs. fixe Dosis) entscheidend.

### Thrombomodulin (TM)

Rekombinantes humanes Thrombomodulin (rhTM), das bislang nur in Japan zur Behandlung der sepsisassoziierten DIC zugelassen ist, zeigte in präklinischen Sepsismodellen antiinflammatorische, antitumorale Aktivität und endothelprotektive Wirkungen [130]. Humanstudien berichteten ebenfalls über Verbesserungen von Zytokinprofilen, Leberfunktion sowie über günstigere Verläufe bei Patienten mit sepsisassoziierter DIC [73, 128, 135]. rhTM moduliert sowohl Immun- als auch Endothelantworten, indem es HMGB1-vermittelte Zellschädigung hemmt, Histone bindet, die NET-Bildung reduziert und Leukozyten-Endothel-Interaktionen unterdrückt [48, 55, 66, 72, 103].

Trotz dieser Daten konnte die multizentrische Phase-III-Studie SCARLET, die den Einsatz von rekombinantem Thrombomodulin auf die 28-Tage-Letalität bei Sepsis untersuchte, keinen Überlebensvorteil für Patienten mit sepsisassoziierter Koagulopathie zeigen; stattdessen traten unter rhTM mehr schwere Blutungen auf als unter Placebo [121]. Post-hoc-Analysen deuten an, dass die Wirksamkeit vom Patiententyp abhängen könnte: Bei persistierender SIC und ohne begleitende Heparintherapie scheinen die Vorteile zu überwiegen [31]. Eine Metaanalyse zeigte eine reduzierte Letalität bei Patienten mit SIC/DIC, jedoch keinen Nutzen bei Patienten ohne Gerinnungsstörung [120].

### Aktiviertes Protein C (aPC)

Niedrige endogene aPC-Spiegel korrelieren mit ausgeprägterer Organdysfunktion [68]. aPC besitzt antikoagulatorische und antiinflammatorische sowie Endothel- bzw. Glykokalyx-protektive Eigenschaften [15, 45, 89, 99, 110, 116].

Rekombinantes aPC (Drotrecogin alfa, aktiviert) verbesserte in Humanstudien die Mikrozirkulation in der schweren Sepsis [24, 26]. Diese Daten zusammen mit der PROWESS-Studie, die eine signifikante Mortalitätsreduktion zeigte, führten 2001 zur Zulassung des Präparats zur Behandlung der schweren Sepsis [8]. Spätere großen Studien, u. a. PROWESS-SHOCK, konnten diesen Überlebensvorteil nicht bestätigen und fanden ein erhöhtes Blutungsrisiko [84]. Drotrecogin alfa wurde 2011 vom Markt genommen.

Seit dem Marktrückzug von Drotrecogin alfa zeigen systematische Übersichtsarbeiten übereinstimmend, dass die bisherige Evidenz nicht ausreicht, um aPC routinemäßig zur Behandlung der schweren Sepsis oder des septischen Schocks zu empfehlen [67, 74]. In den bisherigen Studien wurden weder Ausgangs- noch Zielspiegel des endogenen Protein C berücksichtigt. Die Therapie erfolgte nicht individualisiert, was Überdosierungen wahrscheinlich machte und Blutungen erklären könnte.

Vor diesem Hintergrund bleibt offen, ob Ausgewählte von einer individuell angepassten aPC-basierten Therapie profitieren könnten.

### „Neutrophil extracellular traps“ (NETs)

NETs sind neben der Erregerbekämpfung ein wesentlicher Bestandteil der dysregulierten Immunantwort in den Krankheitsbildern Sepsis und ARDS. Die zellfreie DNA bietet ein Gerüst für die Thrombozytenaggregation und Aktivierung der intrinsischen Gerinnungskaskade [32, 39]. NETs stellen deshalb einen Therapieansatz für die Immunmodulation und Gerinnungsregulation dar. Grundlage für vorrangig experimentelle Therapieansätze bilden dabei die Hauptbestandteile der NETs:

1. Desoxyribonuklease I (DNAse I) löst zirkulierende, zellfreie DNA unspezifisch auf. DNAse I ist als Pulmozyme® bereits als inhalatives Mukolytikum für zystische Fibrose-Patienten zugelassen [122]. Die intravenöse Applikation von DNase I ist bisher nur tierexperimentell untersucht. Hier zeigten sich verbesserte Gerinnungsparameter und ein verbessertes Überleben [76, 87, 138].
2. Histoninhibitoren und Inhibitoren des PAD4-Signalweges, können eine Histoninstabilität und den Zerfall der NETs herbeiführen [79]. Der PAD4-Inhibitor Cl-Amidin zeigte in Sepsismodellen einen antikoagulatorischen Effekt und ein verbessertes Outcome [10, 140]. Enge therapeutische Fenster und eine Zytotoxizität stellen bei vielen PAD4-Inhibitoren eine relevante Limitierung für den klinischen Einsatz dar [80].
3. Auch für die neutrophile Elastase und die Myeloperoxidase existieren Inhibitoren (Sivelestat bzw. PF-1355, Luminol). Eine experimentelle Studie zeigte, dass die alleinige Inhibition der neutrophilen Elastase keine klinisch relevante NET-Reduktion herbeiführt [85].

Die komplette NET-Hemmung schwächt die Immunabwehr stark, sodass eine Translation der bisherigen Erkenntnisse der NET-Hemmung als modulierende Co-Therapie zur antikoagulatorischen und antiinfektiven Therapie diskutiert wird [81].

### Komplement-C5-Inhibition

Präklinische Studien belegen, dass eine C5-Blockade inflammatorische Reaktionen, Endothelschädigung und Koagulopathie reduziert und das Überleben in Modellen bakterieller Sepsis verbessert [28, 33, 40, 43, 96]. Erste klinische Erfahrungen mit C5-Inhibitoren wie Eculizumab oder dem selektiven Anti-C5a-Antikörper Vilobelimab zeigen eine effektive Suppression der C5a-Freisetzung, eine Abschwächung systemischer Inflammation und mögliche Verbesserungen hämodynamischer und respiratorischer Parameter, wenngleich randomisierte Studien bislang keinen konsistenten Mortalitätsvorteil demonstriert haben. Vilobelimab, ein selektiver monoklonaler Anti-C5a-Antikörper, blockiert ausschließlich freies C5a, ohne die Bildung des Membranangriffskomplexes (MAC) zu hemmen. Die komplementvermittelte bakterizide Wirtsabwehr bleibt erhalten [86]. In der Phase-IIa-SCIENS-Studie reduzierte Vilobelimab die C5a-Plasmaspiegel dosisabhängig um bis zu 90 % und war mit mehr ICU-freien und ventilatorfreien Tagen sowie einem rascheren Abfall inflammatorischer Zytokine assoziiert [6]. Jedoch gibt es auch Daten, die darauf hinweisen, dass eine Dauertherapie mit C5-Inhibitoren mit nachteiligen Effekten bzw. einer erhöhten Anfälligkeit für eine Neisserien-Sepsis verbunden sein kann [104].

### Fibrinolyse-Shutdown

Derzeit gibt es keinen Standard für die Erfassung der eingeschränkten Fibrinolyse. Anwendung finden viskoelastische Tests über die maximale Lyse (ML) der intrinsisch bzw. extrinsisch aktivierten Assays. Eine verminderte ML ist jedoch nicht spezifisch für eine reduzierte Fibrinolyse, da auch die thrombozytenvermittelte „Clot Retraction“ (Prozess der Aktin-Myosin-vermittelten Kontraktion des Fibringerinnsels mit konsekutiver Verdichtung und Schrumpfung des Clots sowie Extrusion von Serum) mit einer Abnahme der gemessenen Gerinnselfestigkeit einhergeht. Erst der Vergleich mit Aprotinin- bzw. TXS-Assays, in denen die Plasmin-vermittelte Fibrinolyse pharmakologisch gehemmt wird, ermöglicht die Unterscheidung zwischen einer tatsächlichen Fibrinolyse und einer „Clot Retraction“. Mit den tPA-aktivierten Tests lässt sich die Resistenz gegenüber tPA-induzierter Lyse beurteilen, allerdings ist diese auch von der maximalen Gerinnselfestigkeit (MCF) abhängig und kann bei erhöhter MCF falsch verlängert sein [90]. Dennoch stellt die Hypofibrinolyse in der Sepsis ein unterschätztes Problem dar.

Während der COVID-19-Pandemie wurde versucht den „fibrinolytic shutdown“ durch tPA-Gabe zu behandeln, um die Mikrozirkulation zu verbessern. Bei schwerem COVID-19 entsteht jedoch stark quervernetztes, tPA-resistentes Fibrin, was die Wirksamkeit systemischer Thrombolyse deutlich begrenzt [4]. Bolusgaben führten zu kurzfristigen Verbesserungen der Oxygenierung ohne Überlebensvorteile oder mehr beatmungsfreie Tage, und kontinuierliche Infusionen blieben wirkungslos [4, 75]. Eine Studie, welche mit dem gentechnisch hergestellten tPA Alteplase bei COVID-19-Patienten durchgeführt wurde, zeigte leichte Trends zu besseren Ergebnissen – v. a. bei Hochdosisregimen und nicht intubierten Patienten –, war jedoch nicht schlüssig und wies eine höhere Rate an schweren Blutungen auf [75].

Um die Risiken der systemischen Gabe zu reduzieren, wurde auch inhalatives tPA untersucht. Diese Therapie war gut verträglich und zeigte Tendenzen zur Verbesserung der Oxygenierung, insbesondere bei Patienten mit nichtinvasiver Beatmung, die Evidenz bleibt jedoch vorläufig und basiert nicht auf randomisierten Daten [16]. Insgesamt zeigen tPA-basierte Strategien in COVID-19 und Sepsis zwar biologische Plausibilität, aber nur begrenzte Wirksamkeit – einerseits wegen erhöhter Blutungsgefahr, andererseits, da PAI‑1 auch das exogen verabreichte tPA hemmt. Tenecteplase, das weniger empfindlich gegenüber PAI‑1 ist und eine höhere Fibrinspezifität aufweist, könnte hier Vorteile bieten, obwohl In-vivo-Daten fehlen [102].

Ein weiterer Ansatz ist die Inhibition von PAI‑1, einem Inhibitor der Fibrinolyse. Am vielversprechendsten hierbei ist TM5614 [92]. Allerdings erlauben die bisherigen Humanstudien aufgrund geringer Fallzahlen keine Aussagen zur Wirksamkeit [53]. Beide Studien zeigen eine gute Verträglichkeit, sodass größere Studien sinnvoll und vielversprechend sind [53].

Der direkte Ersatz von Plasminogen erscheint weniger sinnvoll, da ein PAI-1-Überschuss die Aktivierung zu Plasmin verhindert. Plasmin selbst wurde beim Menschen im Rahmen entzündlicher Erkrankungen wie Sepsis bislang nicht eingesetzt; in der Schlaganfalltherapie und in Tiermodellen zeigte es jedoch vielversprechende Resultate [91, 119]. Wegen seiner breiteren Proteolyse besteht ein Risiko für die Lyse anderer Proteine und dem löslichen Fibrinogen, was in einer Hypofibrinogenämie und somit erhöhtem Blutungsrisiko resultieren könnte.

Ein weiterer potenzieller Therapieansatz ist Defibrotid, ein profibrinolytischer endothelialer Modulator, der endogenes tPA erhöht und PAI‑1 über die Beeinflussung des Endothels senkt. Obwohl in Sepsis und Endotheliitis vielversprechend diskutiert, fehlen große klinische Studien. In einer humanen Endotoxämie zeigte Defibrotid eine verstärkte Fibrinolyse und niedrigere CRP-Werte [109].

Ein routinemäßig verfügbarer Wirkstoff, der die Fibrinolyse zuverlässig, aber im geringeren Ausmaß beeinflusst, ist der direkte Thrombininhibitor Argatroban, der zur Therapie der HIT eingesetzt wird. Er erhöht einerseits die Porosität des Fibrinnetzes und hemmt andererseits TAFI effektiver als Heparin, wodurch tPA-induzierte Fibrinolyse erleichtert wird [44, 95]. In tPA-aktivierten Assays zeigt sich dies typischerweise durch geringere MCF und kürzere Lysezeiten.

## Schlussfolgerung

Die SIC ist eine häufige und prognostisch relevante Komplikation der Sepsis, die bei etwa 20–25 % der intensivpflichtigen Patient*innen auftritt und eng mit erhöhter Morbidität und Mortalität assoziiert ist. Für die klinische Praxis ist eine frühzeitige Identifikation der SIC entscheidend. Der ISTH-SIC-Score stellt ein einfach anwendbares Screeninginstrument dar, erlaubt jedoch lediglich eine dichotome Einordnung und weist eine limitierte prognostische Aussagekraft auf. Die Kombination mit etablierten DIC-Scores verbessert die Vorhersage klinischer Endpunkte bislang nicht konsistent. Zudem berücksichtigen viele etablierte Scores primär konventionelle Gerinnungsparameter wie Thrombozytenzahl, Prothrombinzeit oder Fibrinogen im Sinne eines Verbrauchs von Gerinnungsfaktoren. Diese bilden jedoch die für die Sepsis charakteristische immunthrombotische bzw. mikrovaskuläre Thrombenbildung nur unzureichend ab. Dabei ist zu berücksichtigen, dass einzelne Parameter, insbesondere die Thrombozytenzahl, nicht ausschließlich Blutungsrisiken widerspiegeln, sondern im Rahmen der Sepsis auch Ausdruck einer gesteigerten thromboinflammatorischen Aktivität sein können. Eine differenziertere Erfassung pro- und antikoagulatorischer sowie fibrinolytischer Mechanismen erscheint daher pathophysiologisch sinnvoll. In diesem Kontext ermöglichen viskoelastische Testverfahren eine bettseitige, dynamische Beurteilung der Gerinnung, insbesondere der häufig vorliegenden Hypofibrinolyse („fibrinolytic shutdown“), deren Ausmaß mit ungünstigen klinischen Verläufen assoziiert ist, wenngleich die Datenlage insgesamt heterogen bleibt.

**Abb. 3:**
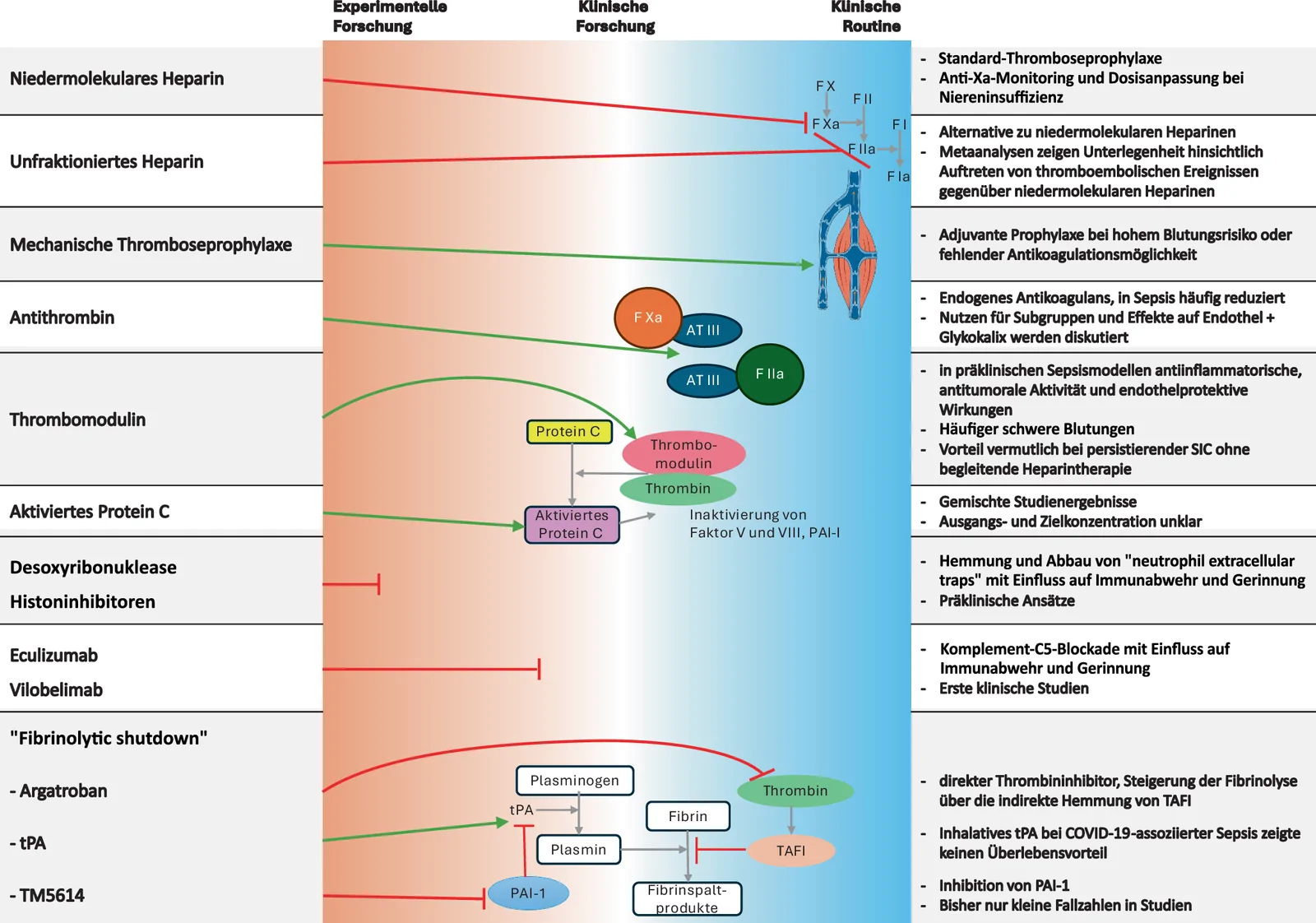
Überblick über verschiedene therapeutische Ansätze der sepsisinduzierten Koagulopathie, unterteilt nach dem jeweiligen Stand der Forschung – von ersten experimentellen Erkenntnissen bis hin zur festen Etablierung in der klinischen Routine. *AT III* Antithrombin, *F* Faktor, *PAI‑1* Plasminogen-Aktivator-Inhibitor 1, *TAFI* Thrombin-aktivierter Fibrinolyseinhibitor, *TF* „tissue factor“, *tPA* „tissue plasminogen activator“. (Die Erlaubnis zur Verwendung der dargestellten Abbildungen erfolgte im Rahmen einer bestehenden Creative Commons 0 1.0 Lizenz [https://creativecommons.org/publicdomain/zero/1.0/] oder einer bestehenden Creative Commons BY 4.0 Lizenz [https://creativecommons.org/licenses/by/4.0/])

Therapeutisch steht die konsequente Thromboseprophylaxe im Vordergrund. NMH sind gegenüber UFH mit einer geringeren Rate venöser Thromboembolien bei vergleichbarem Blutungsrisiko assoziiert, während bei eingeschränkter Nierenfunktion eine differenzierte, häufig UFH-basierte Strategie erforderlich ist. Spezifische gerinnungsmodulierende Therapien wie AT, rTM oder aPC haben in großen randomisierten Studien keinen konsistenten Überlebensvorteil gezeigt und sind derzeit nicht Teil der Therapie; ein potenzieller Nutzen könnte auf selektionierte Patientengruppen mit spezifischen pathophysiologischen Profilen beschränkt sein. Experimentelle Ansätze, die auf NETs, das Komplementsystem oder die Modulation der Fibrinolyse abzielen, erscheinen mechanistisch vielversprechend, bedürfen jedoch weiterer klinischer Evaluation. Die Abb. 3 veranschaulicht die verschiedenen therapeutischen Optionen und den Stand der Entwicklung. Insgesamt erfordert das Management der SIC eine individualisierte, pathophysiologisch orientierte Diagnostik und Therapie.

## Fazit für die Praxis

- Die sepsisinduzierte Koagulopathie (SIC) betrifft etwa 20–25 % der intensivpflichtigen Patient*innen mit Sepsis und ist mit einer ungünstigen Prognose assoziiert.
- Der ISTH-SIC-Score ermöglicht ein standardisiertes Screening, besitzt jedoch nur eine begrenzte prognostische Aussagekraft.
- Konventionelle Gerinnungsparameter erfassen die hämostaseologischen Charakteristika der SIC nur unvollständig; eine funktionelle Beurteilung der Fibrinolyse ermöglichen sie nicht.
- Viskoelastische Tests können die Diagnostik durch eine dynamische Beurteilung der Gerinnselbildung und Fibrinolyse ergänzen; ihr Einsatz zur Erfassung der sepsisassoziierten Hypofibrinolyse ist bislang jedoch nicht hinreichend validiert.
- Die pharmakologische Thromboseprophylaxe bildet eine zentrale Säule des Managements. Niedermolekulare Heparine sind bei fehlenden Kontraindikationen zu bevorzugen; bei schwerer Niereninsuffizienz ist das Akkumulationsrisiko zu berücksichtigen.
- Eine spezifische Therapie der SIC mit Antithrombin, rekombinantem Thrombomodulin oder aktiviertem Protein C ist mangels eines konsistent nachgewiesenen Überlebensvorteils derzeit nicht etabliert.

## Data Availability

Alle dieser Arbeit zugrunde liegenden Daten sind in diesem Artikel enthalten.

## References

[1] Bachler M, Bösch J, Stürzel DP et al. (2021) Impaired fibrinolysis in critically ill COVID-19 patients. British journal of anaesthesia 10.1016/j.bja.2020.12.010 PMC7833514 33422287

[2] Bachler M, Hell T, Schausberger L et al (2019) Response patterns of routinely measured inflammatory and coagulatory parameters in sepsis. PeerJ 7:e7147 10.7717/peerj.7147 PMC6590445 31275752

[3] Badimon L, Vilahur G (2015) Neutrophil extracellular traps: a new source of tissue factor in atherothrombosis. European Heart Journal 36:1364–1366 10.1093/eurheartj/ehv105 25845929

[4] Barrett CD, Moore HB, Moore EE et al (2022) Study of Alteplase for Respiratory Failure in SARS-CoV-2 COVID-19: A Vanguard Multicenter, Rapidly Adaptive, Pragmatic, Randomized Controlled Trial. Chest 161:710–727 10.1016/j.chest.2021.09.024 PMC8474873 34592318

[5] Barrios EL, Rincon JC, Willis M et al (2024) TRANSCRIPTOMIC DIFFERENCES IN PERIPHERAL MONOCYTE POPULATIONS IN SEPTIC PATIENTS BASED ON OUTCOME. Shock 62:208–216 10.1097/SHK.0000000000002379 PMC11892173 38713581

[6] Bauer M, Weyland A, Marx G et al (2021) Efficacy and Safety of Vilobelimab (IFX-1), a Novel Monoclonal Anti-C5a Antibody, in Patients With Early Severe Sepsis or Septic Shock‑A Randomized, Placebo-Controlled, Double-Blind, Multicenter, Phase IIa Trial (SCIENS Study). Crit Care Explor 3:e0577 10.1097/CCE.0000000000000577 PMC8601347 34806021

[7] Belousoviene E, Kiudulaite I, Pilvinis V et al. (2021) Links between Endothelial Glycocalyx Changes and Microcirculatory Parameters in Septic Patients. Life (Basel) 11(8):790 10.3390/life11080790 PMC8398731 34440534

[8] Bernard GR, Vincent JL, Laterre PF et al (2001) Efficacy and safety of recombinant human activated protein C for severe sepsis. The New England journal of medicine 344:699–709 10.1056/NEJM200103083441001 11236773

[9] Beurskens DM, Bol ME, Delhaas T et al (2020) Decreased endothelial glycocalyx thickness is an early predictor of mortality in sepsis. Anaesth Intensive Care 48:221–228 10.1177/0310057X20916471 PMC7328096 32486831

[10] Biron BM, Chung CS, O’brien XM et al (2017) Cl-Amidine Prevents Histone 3 Citrullination and Neutrophil Extracellular Trap Formation, and Improves Survival in a Murine Sepsis Model. J Innate Immun 9:22–32 10.1159/000448808 PMC5219946 27622642

[11] Bode M, Mackman N (2014) Regulation of tissue factor gene expression in monocytes and endothelial cells: Thromboxane A2 as a new player. Vascular pharmacology 62:57–62 10.1016/j.vph.2014.05.005 PMC4116464 24858575

[12] Boscolo A, Spiezia L, De Cassai A et al (2021) Are thromboelastometric and thromboelastographic parameters associated with mortality in septic patients? A systematic review and meta-analysis. J Crit Care 61:5–13 10.1016/j.jcrc.2020.09.034 33049490

[13] Burmeister A, Vidal YSS, Liu X et al (2022) Impact of neutrophil extracellular traps on fluid properties, blood flow and complement activation. Front Immunol 13:1078891 10.3389/fimmu.2022.1078891 PMC9800590 36591269

[14] Carmichael SP 2nd, Appelbaum RD, Renaldo A et al (2023) ENDOTHELIAL GLYCOCALYX SHEDDING IN INTRA-ABDOMINAL SEPSIS: A FEASIBILITY STUDY. Shock 59:540–546 10.1097/SHK.0000000000002079 PMC11213624 36625488

[15] Chen P, Huang L, Zhang Y et al (2010) Anti-apoptotic effect of activated protein C on lipopolysaccharide-stimulated human umbilical vein endothelial cells is associated with the inhibition of the caspase‑3 pathway. Molecular medicine reports 3:991–997 10.3892/mmr.2010.359 21472345

[16] Chowdary P, Agarwal B, Peralta MR et al (2023) Nebulized Recombinant Tissue Plasminogen Activator (rt-PA) for Acute COVID-19-Induced Respiratory Failure: An Exploratory Proof-of-Concept Trial. Journal of Clinical Medicine 12:5848 10.3390/jcm12185848 PMC10531875 37762789

[17] Colicchia M, Perrella G, Gant P et al (2023) Novel mechanisms of thrombo-inflammation during infection: spotlight on neutrophil extracellular trap-mediated platelet activation. Res Pract Thromb Haemost 7:100116 10.1016/j.rpth.2023.100116 PMC10099327 37063765

[18] Coupland LA, Rabbolini DJ, Schoenecker JG et al (2023) Point-of-care diagnosis and monitoring of fibrinolysis resistance in the critically ill: results from a feasibility study. Critical care (London, England) 27:55 10.1186/s13054-023-04329-5 PMC9912243 36765421

[19] Curtiaud A, Iba T, Angles-Cano E et al (2025) Biomarkers of sepsis-induced coagulopathy: diagnostic insights and potential therapeutic implications. Annals of intensive care 15:12 10.1186/s13613-025-01434-2 PMC11739444 39821561

[20] De Vries JJ, Visser C, Geers L et al (2022) Altered fibrin network structure and fibrinolysis in intensive care unit patients with COVID-19, not entirely explaining the increased risk of thrombosis. J Thromb Haemost 20:1412–1420 10.1111/jth.15708 PMC9115158 35316570

[21] Delgadillo LF, Lomakina EB, Kuebel J et al (2021) Changes in endothelial glycocalyx layer protective ability after inflammatory stimulus. Am J Physiol Cell Physiol 320:C216–c224 10.1152/ajpcell.00259.2020 PMC7948006 33326314

[22] Ding R, Wang Z, Lin Y et al (2018) Comparison of a new criteria for sepsis-induced coagulopathy and International Society on Thrombosis and Haemostasis disseminated intravascular coagulation score in critically ill patients with sepsis 3.0: a retrospective study. Blood Coagul Fibrinolysis 29:551–558 10.1097/MBC.0000000000000755 PMC6133197 30015646

[23] Dolmatova EV, Wang K, Mandavilli R et al (2021) The effects of sepsis on endothelium and clinical implications. Cardiovascular research 117:60–73 10.1093/cvr/cvaa070 PMC7810126 32215570

[24] Donati A, Damiani E, Botticelli L et al (2013) The aPC treatment improves microcirculation in severe sepsis/septic shock syndrome. BMC Anesthesiol 13:25 10.1186/1471-2253-13-25 PMC3848895 24070065

[25] Duranteau J, Taccone FS, Verhamme P et al (2018) European guidelines on perioperative venous thromboembolism prophylaxis: Intensive care. Eur J Anaesthesiol 35:142–146 10.1097/EJA.0000000000000707 29112545

[26] Favory R, Poissy J, Alves I et al (2013) Activated protein C improves macrovascular and microvascular reactivity in human severe sepsis and septic shock. Shock 40:512–518 10.1097/SHK.0000000000000060 24052037

[27] Fernández-Sarmiento J, Molina CF, Salazar-Pelaez LM et al (2023) Biomarkers of Glycocalyx Injury and Endothelial Activation are Associated with Clinical Outcomes in Patients with Sepsis: A Systematic Review and Meta-Analysis. Journal of intensive care medicine 38:95–105 10.1177/08850666221109186 35722738

[28] Fernández-Sarmiento J, Schlapbach LJ, Acevedo L et al (2022) Endothelial Damage in Sepsis: The Importance of Systems Biology. Front Pediatr 10:828968 10.3389/fped.2022.828968 PMC8959536 35356443

[29] Fernando SM, Tran A, Cheng W et al (2022) VTE Prophylaxis in Critically Ill Adults: A Systematic Review and Network Meta-analysis. Chest 161:418–428 10.1016/j.chest.2021.08.050 34419428

[30] Folco EJ, Mawson TL, Vromman A et al (2018) Neutrophil Extracellular Traps Induce Endothelial Cell Activation and Tissue Factor Production Through Interleukin-1α and Cathepsin G. Arterioscler Thromb Vasc Biol 38:1901–1912 10.1161/ATVBAHA.118.311150 PMC6202190 29976772

[31] François B, Fiancette M, Helms J et al (2021) Efficacy and safety of human soluble thrombomodulin (ART-123) for treatment of patients in France with sepsis-associated coagulopathy: post hoc analysis of SCARLET. Annals of intensive care 11:53 10.1186/s13613-021-00842-4 PMC8012451 33788052

[32] Fuchs TA, Brill A, Duerschmied D et al (2010) Extracellular DNA traps promote thrombosis. Proceedings of the National Academy of Sciences 107:15880–15885 10.1073/pnas.1005743107 PMC2936604 20798043

[33] Furebring M, Håkansson LD, Venge P et al (2002) Expression of the C5a receptor (CD88) on granulocytes and monocytes in patients with severe sepsis. Critical care (London, England) 6:363–370 10.1186/cc1524 PMC125318 12225614

[34] Gajsiewicz JM, Smith SA, Morrissey JH (2017) Polyphosphate and RNA Differentially Modulate the Contact Pathway of Blood Clotting. J Biol Chem 292:1808–1814 10.1074/jbc.M116.754325 PMC5290954 28007958

[35] Gander-Bui HTT, Schläfli J, Baumgartner J et al (2023) Targeted removal of macrophage-secreted interleukin‑1 receptor antagonist protects against lethal Candida albicans sepsis. Immunity 56:1743–1760.e1749 10.1016/j.immuni.2023.06.023 37478856

[36] Gando S, Saitoh D, Ishikura H et al (2013) A randomized, controlled, multicenter trial of the effects of antithrombin on disseminated intravascular coagulation in patients with sepsis. Critical care (London, England) 17:R297 10.1186/cc13163 PMC4057033 24342495

[37] Gando S, Wada H, Asakura H et al (2005) Evaluation of new Japanese diagnostic criteria for disseminated intravascular coagulation in critically ill patients. Clin Appl Thromb Hemost 11:71–76 10.1177/107602960501100108 15678275

[38] Gouin-Thibault I, Mansour A, Caribotti C et al (2024) Tinzaparin, an alternative to subcutaneous unfractionated heparin, in patients with severe and end-stage renal impairment: a retrospective observational single-center study. J Thromb Haemost 22:2864–2872 10.1016/j.jtha.2024.07.006 39019439

[39] Gould TJ, Vu TT, Swystun LL et al (2014) Neutrophil extracellular traps promote thrombin generation through platelet-dependent and platelet-independent mechanisms. Arterioscler Thromb Vasc Biol 34:1977–1984 10.1161/ATVBAHA.114.304114 25012129

[40] Guglietta S, Chiavelli A, Zagato E et al (2016) Coagulation induced by C3aR-dependent NETosis drives protumorigenic neutrophils during small intestinal tumorigenesis. Nat Commun 7:11037 10.1038/ncomms11037 PMC4802169 26996437

[41] Hatanaka K, Ito T, Madokoro Y et al (2021) Circulating Syndecan‑1 as a Predictor of Persistent Thrombocytopenia and Lethal Outcome: A Population Study of Patients With Suspected Sepsis Requiring Intensive Care. Front Cardiovasc Med 8:730553 10.3389/fcvm.2021.730553 PMC8452900 34557532

[42] Haykal T, Zayed Y, Dhillon H et al (2022) Meta-Analysis of the Role of Intermittent Pneumatic Compression of the Lower Limbs to Prevent Venous Thromboembolism in Critically Ill Patients. Int J Low Extrem Wounds 21:31–40 10.1177/1534734620925391 32527203

[43] Hazelzet JA, De Groot R, Van Mierlo G et al (1998) Complement activation in relation to capillary leakage in children with septic shock and purpura. Infect Immun 66:5350–5356 10.1128/iai.66.11.5350-5356.1998 PMC108669 9784543

[44] He S, Blombäck M, Bark N et al (2010) The direct thrombin inhibitors (argatroban, bivalirudin and lepirudin) and the indirect Xa-inhibitor (danaparoid) increase fibrin network porosity and thus facilitate fibrinolysis. Thromb Haemost 103:1076–1084 10.1160/TH09-05-0306 20216982

[45] Healy LD, Puy C, Fernández JA et al (2017) Activated protein C inhibits neutrophil extracellular trap formation in vitro and activation in vivo. J Biol Chem 292:8616–8629 10.1074/jbc.M116.768309 PMC5448091 28408624

[46] Helms J, Iba T, Connors JM et al (2023) How to manage coagulopathies in critically ill patients. Intensive care medicine 49:273–290 10.1007/s00134-023-06980-6 36808215

[47] Helms J, Severac F, Merdji H et al (2020) Performances of disseminated intravascular coagulation scoring systems in septic shock patients. Annals of intensive care 10:92 10.1186/s13613-020-00704-5 PMC7352012 32651674

[48] Herzog C, Lorenz A, Gillmann HJ et al (2014) Thrombomodulin’s lectin-like domain reduces myocardial damage by interfering with HMGB1-mediated TLR2 signalling. Cardiovascular research 101:400–410 10.1093/cvr/cvt275 24323314

[49] Heubner L, Greiner M, Vicent O et al (2022) Predictive ability of viscoelastic testing using ClotPro® for short-term outcome in patients with severe Covid-19 ARDS with or without ECMO therapy: a retrospective study. Thromb J 20:48 10.1186/s12959-022-00403-0 PMC9421107 36038895

[50] Heubner L, Mirus M, Vicent O et al. (2022) Point of care coagulation management in anesthesiology and critical care. Minerva Anestesiol 88(7–8):615–628 10.23736/S0375-9393.22.16380-7 35416466

[51] Heurich M, Mccluskey G (2023) Complement and coagulation crosstalk - Factor H in the spotlight. Immunobiology 228:152707 10.1016/j.imbio.2023.152707 37633063

[52] Hippensteel JA, Uchimido R, Tyler PD et al (2019) Intravenous fluid resuscitation is associated with septic endothelial glycocalyx degradation. Critical care (London, England) 23:259 10.1186/s13054-019-2534-2 PMC6652002 31337421

[53] Hirai T, Asano K, Ito I et al (2024) A randomized double-blind placebo-controlled trial of an inhibitor of plasminogen activator inhibitor‑1 (TM5614) in mild to moderate COVID-19. Scientific reports 14:165 10.1038/s41598-023-50445-1 PMC10761996 38168544

[54] Huang X, Hu H, Sun T et al (2021) Plasma Endothelial Glycocalyx Components as a Potential Biomarker for Predicting the Development of Disseminated Intravascular Coagulation in Patients With Sepsis. Journal of intensive care medicine 36:1286–1295 10.1177/0885066620949131 32799720

[55] Iba T, Aihara K, Watanabe S et al (2013) Recombinant thrombomodulin improves the visceral microcirculation by attenuating the leukocyte-endothelial interaction in a rat LPS model. Thrombosis research 131:295–299 10.1016/j.thromres.2012.11.025 23273403

[56] Iba T, Nisio MD, Levy JH et al (2017) New criteria for sepsis-induced coagulopathy (SIC) following the revised sepsis definition: a retrospective analysis of a nationwide survey. BMJ Open 7:e017046 10.1136/bmjopen-2017-017046 PMC5623518 28963294

[57] Iba T, Di Nisio M, Thachil J et al (2018) A Proposal of the Modification of Japanese Society on Thrombosis and Hemostasis (JSTH) Disseminated Intravascular Coagulation (DIC) Diagnostic Criteria for Sepsis-Associated DIC. Clin Appl Thromb Hemost 24:439–445 10.1177/1076029617720069 PMC6714647 28747069

[58] Iba T, Levi M, Thachil J et al (2023) Communication from the Scientific and Standardization Committee of the International Society on Thrombosis and Haemostasis on sepsis-induced coagulopathy in the management of sepsis. J Thromb Haemost 21:145–153 10.1016/j.jtha.2022.10.022 36695377

[59] Iba T, Maier CL, Tanigawa T et al (2023) Risk stratification utilizing sequential organ failure assessment (SOFA) score, antithrombin activity, and demographic data in sepsis-associated disseminated intravascular coagulation (DIC). Scientific reports 13:22502 10.1038/s41598-023-49855-y PMC10728127 38110515

[60] Iba T et al. J Thromb Haemost. 2025;23:2356–2362. 10.1016/j.jtha.2025.03.038 40216223

[61] Inkinen N, Pettilä V, Lakkisto P et al (2019) Association of endothelial and glycocalyx injury biomarkers with fluid administration, development of acute kidney injury, and 90-day mortality: data from the FINNAKI observational study. Annals of intensive care 9:103 10.1186/s13613-019-0575-y PMC6738365 31512003

[62] Ishikawa M, Yamashita H, Oka N et al (2017) Antithrombin III improved neutrophil extracellular traps in lung after the onset of endotoxemia. J Surg Res 208:140–150 10.1016/j.jss.2016.09.041 27993201

[63] Jans J, Van Dun SCJ, Gorissen R et al (2024) The monocyte-derived cytokine response in whole blood from preterm newborns against sepsis-related bacteria is similar to term newborns and adults. Front Immunol 15:1353039 10.3389/fimmu.2024.1353039 PMC10982322 38562936

[64] Jiang M, Wu W, Xia Y et al (2023) Platelet-derived extracellular vesicles promote endothelial dysfunction in sepsis by enhancing neutrophil extracellular traps. BMC immunology 24:22 10.1186/s12865-023-00560-5 PMC10413488 37559007

[65] Joffre J, Hellman J (2021) Oxidative Stress and Endothelial Dysfunction in Sepsis and Acute Inflammation. Antioxid Redox Signal 35:1291–1307 10.1089/ars.2021.0027 33637016

[66] Kajioka H, Kagawa S, Ito A et al (2021) Targeting neutrophil extracellular traps with thrombomodulin prevents pancreatic cancer metastasis. Cancer letters 497:1–13 10.1016/j.canlet.2020.10.015 33065249

[67] Kalil AC, Larosa SP (2012) Effectiveness and safety of drotrecogin alfa (activated) for severe sepsis: a meta-analysis and metaregression. Lancet Infect Dis 12:678–686 10.1016/S1473-3099(12)70157-3 22809883

[68] Katayama S, Koyama K, Shima J et al (2019) Thrombomodulin, Plasminogen Activator Inhibitor‑1 and Protein C Levels, and Organ Dysfunction in Sepsis. Crit Care Explor 1:e0013 10.1097/CCE.0000000000000013 PMC7063963 32166258

[69] Kienast J, Juers M, Wiedermann CJ et al (2006) Treatment effects of high-dose antithrombin without concomitant heparin in patients with severe sepsis with or without disseminated intravascular coagulation. J Thromb Haemost 4:90–97 10.1111/j.1538-7836.2005.01697.x 16409457

[70] Koami H, Sakamoto Y, Hirota Y et al (2025) Effect of hypofibrinolysis on clinical outcomes of patients with septic disseminated intravascular coagulation. Thrombosis research 245:109235 10.1016/j.thromres.2024.109235 39644620

[71] Kruse JM, Magomedov A, Kurreck A et al (2020) Thromboembolic complications in critically ill COVID-19 patients are associated with impaired fibrinolysis. Critical Care 24:676 10.1186/s13054-020-03401-8 PMC7719734 33287877

[72] Kudo D, Toyama M, Aoyagi T et al (2013) Involvement of high mobility group box 1 and the therapeutic effect of recombinant thrombomodulin in a mouse model of severe acute respiratory distress syndrome. Clinical and experimental immunology 173:276–287 10.1111/cei.12106 PMC3722928 23607598

[73] Kurokohchi K, Imataki O, Kubo F (2015) Anti-inflammatory effect of recombinant thrombomodulin for fulminant hepatic failure. World journal of gastroenterology 21:8203–8207 10.3748/wjg.v21.i26.8203 PMC4499366 26185395

[74] Lai PS, Matteau A, Iddriss A et al (2013) An updated meta-analysis to understand the variable efficacy of drotrecogin alfa (activated) in severe sepsis and septic shock. Minerva Anestesiol 79:33–43 PMC3725305 23174922

[75] Landoni G, Chowdary P, Meziani F et al (2024) Alteplase in COVID-19 severe hypoxemic respiratory failure: the TRISTARDS multicenter randomized trial. Annals of intensive care 14:170 10.1186/s13613-024-01386-z PMC11551089 39522090

[76] Lauková L, Konečná B, Bábíčková J et al (2017) Exogenous deoxyribonuclease has a protective effect in a mouse model of sepsis. Biomed Pharmacother 93:8–16 10.1016/j.biopha.2017.06.009 28622595

[77] Levi M, Poll T (2015) Coagulation in patients with severe sepsis. Semin Thromb Hemost 41:9–15 10.1055/s-0034-1398376 25590524

[78] Levi M, Toh CH, Thachil J et al (2009) Guidelines for the diagnosis and management of disseminated intravascular coagulation. British Committee for Standards in Haematology. Br J Haematol 145:24–33 10.1111/j.1365-2141.2009.07600.x 19222477

[79] Li J, Geng Y, Luo Y et al (2025) Pathological roles of NETs-platelet synergy in thrombotic diseases: From molecular mechanisms to therapeutic targeting. Int Immunopharmacol 159:114934 10.1016/j.intimp.2025.114934 40418882

[80] Li L, Huang HC, He Y et al (2025) Cell-free DNA in sepsis: from molecular insights to clinical management. Mil Med Res 12:85 10.1186/s40779-025-00668-2 PMC12670778 41327452

[81] Liao F, Fan J, Wang R et al (2025) Neutrophil extracellular traps in sepsis: trade-off between pros and cons. Burns Trauma 13:tkaf046 10.1093/burnst/tkaf046 PMC12403064 40904580

[82] Locke M, Longstaff C (2021) Extracellular Histones Inhibit Fibrinolysis through Noncovalent and Covalent Interactions with Fibrin. Thromb Haemost 121:464–476 10.1055/s-0040-1718760 PMC7982298 33131044

[83] Lyons PG, Micek ST, Hampton N et al (2018) Sepsis-Associated Coagulopathy Severity Predicts Hospital Mortality*. Critical care medicine 46:736–742 10.1097/CCM.0000000000002997 29373360

[84] Martí-Carvajal AJ, Solà I, Gluud C et al (2012) Human recombinant protein C for severe sepsis and septic shock in adult and paediatric patients. Cochrane Database Syst Rev 12:Cd004388 10.1002/14651858.CD004388.pub6 PMC6464614 23235609

[85] Martinod K, Witsch T, Farley K et al (2016) Neutrophil elastase-deficient mice form neutrophil extracellular traps in an experimental model of deep vein thrombosis. J Thromb Haemost 14:551–558 10.1111/jth.13239 PMC4785059 26712312

[86] Mccarthy MW, Chong C, Riedemann NC et al (2025) A Targeted Blockade of Terminal C5a Is Critical to Management of Sepsis and Acute Respiratory Distress Syndrome: The Mechanism of Action of Vilobelimab. International Journal of Molecular Sciences 26:9628 10.3390/ijms26199628 PMC12524425 41096893

[87] Medeiros SK, Sharma N, Dwivedi D et al (2023) THE EFFECTS OF DNASE I AND LOW-MOLECULAR-WEIGHT HEPARIN IN A MURINE MODEL OF POLYMICROBIAL ABDOMINAL SEPSIS. Shock 59:666–672 10.1097/SHK.0000000000002095 36852972

[88] Mihajlovic DM, Lendak DF, Draskovic BG et al (2015) Thrombomodulin is a Strong Predictor of Multiorgan Dysfunction Syndrome in Patients With Sepsis. Clin Appl Thromb Hemost 21:469–474 10.1177/1076029613508600 24203354

[89] Minhas N, Xue M, Jackson CJ (2017) Activated protein C binds directly to Tie2: possible beneficial effects on endothelial barrier function. Cellular and molecular life sciences : CMLS 74:1895–1906 10.1007/s00018-016-2440-6 PMC11107519 28005151

[90] Mirus M, Schöchl H, Groene P et al. (2025) [Point of care coagulation diagnostics-Absolutely necessary or only a reasonable supplement?]. Anaesthesiologie 74(5):264–274 10.1007/s00101-025-01524-7 40172631

[91] Mitchell PJ, Yan B, Brozman M et al (2017) Plasmin (Human) Administration in Acute Middle Cerebral Artery Ischemic Stroke: Phase 1/2a, Open-Label, Dose-Escalation, Safety Study. J Stroke Cerebrovasc Dis 26:308–320 10.1016/j.jstrokecerebrovasdis.2016.09.022 27793534

[92] Miyata T (2025) Overview: PAI-1 inhibitors and clinical applications. 2026, Biomed J 49(1):100874; online bereits 2025 erschienen 10.1016/j.bj.2025.100874 PMC12859776 40403845

[93] Musgrave KM, Scott J, Sendama W et al (2023) Tissue factor expression in monocyte subsets during human immunothrombosis, endotoxemia and sepsis. Thrombosis research 228:10–20 10.1016/j.thromres.2023.05.018 37263122

[94] Niederwanger C, Hell T, Hofer S et al (2018) Antithrombin deficiency is associated with mortality and impaired organ function in septic pediatric patients: a retrospective study. PeerJ 6:e5538 10.7717/peerj.5538 PMC6129139 30202654

[95] Nielsen VG, Kirklin JK (2008) Argatroban enhances fibrinolysis by differential inhibition of thrombin-mediated activation of thrombin activatable fibrinolysis inhibitor and factor XIII. Blood Coagul Fibrinolysis 19:793–800 10.1097/MBC.0b013e328317f5aa 19002046

[96] Opal SM, Van Der Poll T (2015) Endothelial barrier dysfunction in septic shock. J Intern Med 277:277–293 10.1111/joim.12331 25418337

[97] Ostrowski SR, Haase N, Muller RB et al (2015) Association between biomarkers of endothelial injury and hypocoagulability in patients with severe sepsis: a prospective study. Critical care (London, England) 19:191 10.1186/s13054-015-0918-5 PMC4423170 25907781

[98] Panigada M, Spinelli E, De Falco S et al (2022) The relationship between antithrombin administration and inflammation during veno-venous ECMO. Scientific reports 12:14284 10.1038/s41598-022-17227-7 PMC9395326 35995816

[99] Park SW, Chen SW, Kim M et al (2009) Human activated protein C attenuates both hepatic and renal injury caused by hepatic ischemia and reperfusion injury in mice. Kidney Int 76:739–750 10.1038/ki.2009.255 19625989

[100] Pfeiler S, Stark K, Massberg S et al (2017) Propagation of thrombosis by neutrophils and extracellular nucleosome networks. Haematologica 102:206–213 10.3324/haematol.2016.142471 PMC5286929 27927771

[101] Piotti A, Novelli D, Meessen J et al (2021) Endothelial damage in septic shock patients as evidenced by circulating syndecan‑1, sphingosine-1-phosphate and soluble VE-cadherin: a substudy of ALBIOS. Critical care (London, England) 25:113 10.1186/s13054-021-03545-1 PMC7980645 33741039

[102] Poor H, Yaeger K, Deeba S et al (2024) Tenecteplase With Concomitant Anticoagulation for Acute Respiratory Failure in Patients With COVID-19: A Randomized Controlled Trial. Cureus 16:e54298 10.7759/cureus.54298 PMC10944634 38496180

[103] Rajashekhar G, Gupta A, Marin A et al (2012) Soluble thrombomodulin reduces inflammation and prevents microalbuminuria induced by chronic endothelial activation in transgenic mice. American journal of physiology. Renal physiology 302:F703–F712 10.1152/ajprenal.00558.2011 PMC3311320 22129968

[104] Reher D, Fuhrmann V, Kluge S et al (2018) A rare case of septic shock due to Neisseria meningitidis serogroup B infection despite prior vaccination in a young adult with paroxysmal nocturnal haemoglobinuria receiving eculizumab. Vaccine 36:2507–2509 10.1016/j.vaccine.2018.03.087 29631884

[105] Saoraya J, Wongsamita L, Srisawat N et al (2021) Plasma syndecan‑1 is associated with fluid requirements and clinical outcomes in emergency department patients with sepsis. Am J Emerg Med 42:83–89 10.1016/j.ajem.2021.01.019 33493833

[106] Schmoch T, Brenner T, Becker-Pennrich A et al (2021) Therapie der sepsisinduzierten Koagulopathie. Der Anaesthesist 70:662–670 10.1007/s00101-021-00916-9 PMC7869761 33558944

[107] Schmoch T, Möhnle P, Nusshag C et al. (2025) Impending sepsis-induced coagulopathy (SIC) is associated with increased disease severity in SIC-negative patients: a secondary analysis of a prospective exploratory study. Frontiers in Medicine 12:1525538 10.3389/fmed.2025.1525538 PMC11873100 40034384

[108] Schmoch T, Möhnle P, Weigand MA et al (2023) The prevalence of sepsis-induced coagulopathy in patients with sepsis – a secondary analysis of two German multicenter randomized controlled trials. Annals of intensive care 13:3 10.1186/s13613-022-01093-7 PMC9837358 36635426

[109] Schmoch T, Möhnle P, Weigand MA et al. (2025) Incidence of Sepsis-Induced Coagulopathy (INSIC) Trial: Study Protocol. J Clin Med 14(12):4222. 10.3390/jcm14124222 PMC12194450 40565967

[110] Schuepbach RA, Feistritzer C, Fernández JA et al (2009) Protection of vascular barrier integrity by activated protein C in murine models depends on protease-activated receptor‑1. Thromb Haemost 101:724–733 10.1160/th08-10-0632 PMC2680237 19350118

[111] Semeraro F, Ammollo CT, Morrissey JH et al (2011) Extracellular histones promote thrombin generation through platelet-dependent mechanisms: involvement of platelet TLR2 and TLR4. Blood 118:1952–1961 10.1182/blood-2011-03-343061 PMC3158722 21673343

[112] Shaw AD, Vail GM, Haney DJ et al (2011) Severe protein C deficiency is associated with organ dysfunction in patients with severe sepsis. J Crit Care 26:539–545 10.1016/j.jcrc.2011.05.006 21737232

[113] Singer M, Deutschman CS, Seymour CW et al (2016) The Third International Consensus Definitions for Sepsis and Septic Shock (Sepsis-3). Jama 315:801–810 10.1001/jama.2016.0287 PMC4968574 26903338

[114] Skendros P, Mitsios A, Chrysanthopoulou A et al (2020) Complement and tissue factor-enriched neutrophil extracellular traps are key drivers in COVID-19 immunothrombosis. J Clin Invest 130:6151–6157 10.1172/JCI141374 PMC7598040 32759504

[115] Soubihe N Neto, De Almeida MCV, Couto HO et al (2024) Biomarkers of endothelial glycocalyx damage are associated with microvascular dysfunction in resuscitated septic shock patients. Microvasc Res 154:104683 10.1016/j.mvr.2024.104683 38522507

[116] Tarhan OR, Ceylan BG, Kapucuoglu N et al (2011) Activated protein C and normal saline infusion might prevent deleterious effects of remote acute lung injury caused by intestinal ischemia-reperfusion: an experimental study in the rat model. J Surg Res 168:e81–e86 10.1016/j.jss.2009.12.002 20338588

[117] Taylor FB Jr., Toh CH, Hoots WK et al (2001) Towards definition, clinical and laboratory criteria, and a scoring system for disseminated intravascular coagulation. Thromb Haemost 86:1327–1330 11816725

[118] Tuan TA, Ha NTT, Xoay TD et al (2021) Fibrinolytic Impairment and Mortality in Pediatric Septic Shock: A Single-Center Prospective Observational Study. Pediatr Crit Care Med 22:969–977 10.1097/PCC.0000000000002759 33965991

[119] Vago JP, Zaidan I, Perucci LO et al. (2023) Plasmin and plasminogen prevent sepsis severity by reducing neutrophil extracellular traps and systemic inflammation. JCI Insight 8(8):e166044 10.1172/jci.insight.166044 PMC10243804 36917195

[120] Valeriani E, Squizzato A, Gallo A et al (2020) Efficacy and safety of recombinant human soluble thrombomodulin in patients with sepsis-associated coagulopathy: A systematic review and meta-analysis. J Thromb Haemost 18:1618–1625 10.1111/jth.14812 32237269

[121] Vincent JL, Francois B, Zabolotskikh I et al (2019) Effect of a Recombinant Human Soluble Thrombomodulin on Mortality in Patients With Sepsis-Associated Coagulopathy: The SCARLET Randomized Clinical Trial. Jama 321:1993–2002 10.1001/jama.2019.5358 PMC6547077 31104069

[122] Wagener JS, Kupfer O (2012) Dornase alfa (Pulmozyme). Curr Opin Pulm Med 18:609–614 10.1097/MCP.0b013e328358d51f 22990660

[123] Williams B, Zou L, Pittet JF (2024) Sepsis-Induced Coagulopathy: A Comprehensive Narrative Review of Pathophysiology, Clinical Presentation, Diagnosis, and Management Strategies. Anesth Analg 138(4):696–711 10.1213/ANE.0000000000006888 PMC10916756 38324297

[124] Wang Y, Huang D, Wang M et al (2020) Can Intermittent Pneumatic Compression Reduce the Incidence of Venous Thrombosis in Critically Ill Patients: A Systematic Review and Meta-Analysis. Clin Appl Thromb Hemost 26:1076029620913942 10.1177/1076029620913942 PMC7592327 33074726

[125] Warren BL, Eid A, Singer P et al (2001) Caring for the critically ill patient. High-dose antithrombin III in severe sepsis: a randomized controlled trial. Jama 286:1869–1878 10.1001/jama.286.15.1869 11597289

[126] Whitefoot-Keliin KM, Benaske CC, Allen ER et al. (2024) In response to bacteria, neutrophils release extracellular vesicles capable of initiating thrombin generation through DNA-dependent and independent pathways. J Leukoc Biol 116(6):1223–1236 10.1093/jleuko/qiae125 PMC11599124 38809773

[127] Xue M, Sun Z, Shao M et al (2015) Diagnostic and prognostic utility of tissue factor for severe sepsis and sepsis-induced acute lung injury. J Transl Med 13:172–172 10.1186/s12967-015-0518-9 PMC4459056 26025445

[128] Yamakawa K, Fujimi S, Mohri T et al (2011) Treatment effects of recombinant human soluble thrombomodulin in patients with severe sepsis: a historical control study. Critical care (London, England) 15:R123 10.1186/cc10228 PMC3218981 21569368

[129] Yamakawa K, Umemura Y, Mochizuki K et al (2024) Proposal and Validation of a Clinically Relevant Modification of the Japanese Association for Acute Medicine Disseminated Intravascular Coagulation Diagnostic Criteria for Sepsis. Thromb Haemost 124:1003–1012 10.1055/s-0044-1786808 PMC11518615 38729189

[130] Yang J, Gu Y, Wang T et al (2025) The Multifaceted Roles of Thrombomodulin: Anti-coagulation, Anti-inflammation, and Anti-tumor Potential. Curr Pharm Des 31:1673–1682 10.2174/0113816128335289241218161938 39901541

[131] Yang X, Li L, Liu J et al (2016) Extracellular histones induce tissue factor expression in vascular endothelial cells via TLR and activation of NF-κB and AP‑1. Thrombosis research 137:211–218 10.1016/j.thromres.2015.10.012 26476743

[132] Yasuda N, Goto K, Ohchi Y et al (2016) The efficacy and safety of antithrombin and recombinant human thrombomodulin combination therapy in patients with severe sepsis and disseminated intravascular coagulation. J Crit Care 36:29–34 10.1016/j.jcrc.2016.06.008 27546744

[133] Yen YT, Yang HR, Lo HC et al (2013) Enhancing autophagy with activated protein C and rapamycin protects against sepsis-induced acute lung injury. Surgery 153:689–698 10.1016/j.surg.2012.11.021 23434181

[134] Yong J, Abrams ST, Wang G et al (2023) Cell-free histones and the cell-based model of coagulation. J Thromb Haemost 21:1724–1736 10.1016/j.jtha.2023.04.018 37116754

[135] Yoshihiro S, Sakuraya M, Hayakawa M et al (2019) Recombinant Human-Soluble Thrombomodulin Contributes to Reduced Mortality in Sepsis Patients With Severe Respiratory Failure: A Retrospective Observational Study Using a Multicenter Dataset. Shock 51:174–179 10.1097/SHK.0000000000001148 PMC6319596 29596106

[136] Zhang C, Zhang Z, Mi J et al (2019) The cumulative venous thromboembolism incidence and risk factors in intensive care patients receiving the guideline-recommended thromboprophylaxis. Medicine (Baltimore) 98:e15833 10.1097/MD.0000000000015833 PMC6571428 31169685

[137] Zhang H, Zhou Y, Qu M et al (2021) Tissue Factor-Enriched Neutrophil Extracellular Traps Promote Immunothrombosis and Disease Progression in Sepsis-Induced Lung Injury. Front Cell Infect Microbiol 11:677902 10.3389/fcimb.2021.677902 PMC8317465 34336711

[138] Zhang M, Yan W, Wang T et al (2024) Deoxyribonuclease I Alleviates Septic Liver Injury in a Rat Model Supported by Venoarterial Extracorporeal Membrane Oxygenation. Asaio j 70:241–247 10.1097/MAT.0000000000002084 PMC10885865 37923309

[139] Zhang S, Zhang N, Han J et al (2024) Dynamic immune status analysis of peripheral blood mononuclear cells in patients with Klebsiella pneumoniae bloodstream infection sepsis using single-cell RNA sequencing. Front Immunol 15:1380211 10.3389/fimmu.2024.1380211 PMC11185935 38898888

[140] Zhao T, Pan B, Alam HB et al (2016) Protective effect of Cl-amidine against CLP-induced lethal septic shock in mice. Scientific reports 6:36696 10.1038/srep36696 PMC5098180 27819302

[141] Zhi F, Ma JW, Ji DD et al (2024) Causal associations between circulating cytokines and risk of sepsis and related outcomes: a two-sample Mendelian randomization study. Front Immunol 15:1336586 10.3389/fimmu.2024.1336586 PMC10948396 38504987

